# Quantum indeterminacy: a matter of degree?

**DOI:** 10.1007/s13194-025-00637-5

**Published:** 2025-03-01

**Authors:** Maria Nørgaard

**Affiliations:** Department of Philosophy, University of Geneva, Geneva, Switzerland

**Keywords:** Quantum mechanics, Indeterminacy, Location, Quantum value indefiniteness, Graded properties

## Abstract

The degreed view is an influential account in the debate on quantum value indefiniteness, linking the gradedness of quantum properties to quantum indeterminacy. This paper challenges the connection between degrees and indeterminacy by presenting an example of a graded quantum property that does not entail metaphysical indeterminacy. Through an investigation of two graded approaches to location in quantum mechanics, the paper argues that while the first account, degreed instantiation of *exact location*, is indeterminate, the second account, degreed *quantum location*, is not. This indicates that indeterminacy and degrees are not inherently linked, offering a novel perspective on quantum ontology. The paper advocates adopting a graded view of quantum properties without indeterminacy, potentially leading to a paradigm shift in understanding quantum phenomena.

The emergence of quantum value indefiniteness has led to a flourishing of metaphysical views accounting for the imprecise nature of quantum phenomena, with one of the most influential approaches utilising metaphysical indeterminacy to model quantum value indefiniteness. In particular, a substantial body of literature (Wilson, 2013; Calosi & Wilson, 2019; Ney, 2020; Calosi & Wilson, 2021; Calosi & Mariani, 2021) has focused on the degreed view, positing that quantum indeterminacy stems from the graded nature of quantum properties. Central to this view is the presumed connection between properties coming in degrees and metaphysical indeterminacy.^1^ This paper challenges this connection by providing an example of a graded quantum property that does not give rise to quantum indeterminacy. The analysis provides evidence to support that degrees and indeterminacy come apart: while quantum phenomena may be best captured in degrees, this does not necessitate a metaphysically indeterminate quantum realm. Overall, these findings challenge the current understanding of quantum indeterminacy, motivating a potential shift in the approach to graded quantum ontology.

The paper is structured as follows. In Section 1, I introduce a metaphysics of graded properties, distinguishing the *degreed instantiation* view from the *degreed property* view. In Section 2, I apply these distinct views to quantum ontology, providing different graded accounts of the location of quantum mechanical systems. I proceed using *exact location* and *quantum location* (Nørgaard, 2025) as case studies, arguing that the former is best suited for the degreed instantiation approach, while the latter favours the degreed property view of graded metaphysics. In Section 3, I investigate how these graded accounts are evaluated under two interpretations of indeterminacy: the truth-value gap approach (Torza, 2020; Fletcher & Taylor, 2021) and the determinable-approach (Wilson, 2013; Calosi & Wilson, 2019). I argue that while degreed instantiation of *exact location* is indeterminate on both interpretations of indeterminacy, the degreed *quantum location* account is indeterminate on neither. In Section 4, I conclude that *quantum location* provides a graded account of quantum location without a commitment to metaphysical indeterminacy.

## Metaphysics of graded properties

Metaphysical accounts of graded qualities dispute the orthodox metaphysical claim that property attribution is an *all-or-nothing* matter and allow for the attribution of graded properties instead. In this view, an object has properties not just simpliciter but rather to some degree. At first glance, the idea of graded qualities may appear metaphysically obscure, for what does it mean to have a property only to a degree? Although controversial, several philosophers have argued for the metaphysical intelligibility of an ontology of graded properties (Rosen & Smith, 2004; Myrvold, 2017; Calosi & Wilson, 2019; Ney, 2020; Calosi & Michels, 2025). In this section, I introduce a significant distinction within the possible ontology of graded properties: the distinction between the *degreed instantiation* and the *degreed properties* approach. To understand the significance of the graded approach, consider first the default orthodox view on the metaphysics of properties.

### The orthodox view

According to the orthodox view of properties, the instantiation of a property is an absolute matter: objects either do or do not have properties. The account may be characterised as follows:

the orthodox view: a system *s* is attributed a(n absolute) property *P*, if, and only if, *s* (absolutely) instantiates *P*.

**Fig. 1:**
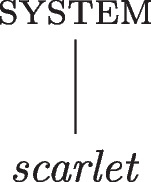
The absolute instantiation of the colour *scarlet*. The vertical line represents the instantiation of the property by the system

This characterisation of the orthodox view is ontologically committed to at least two types of entities: objects and properties.^2^ The *absoluteness* of instantiation in the orthodox view signifies the all-or-nothing nature of instantiation: an object either does (or does not) have a property since an object either does (or does not) instantiate the relevant property. This absoluteness of properties and instantiation is not usually specified since it is an underlying assumption in the metaphysical tradition. In other words, the orthodox account of property instantiation is an uncomplicated matter; there is no metaphysical vagueness or in-betweenness (see Fig. 1 for an illustration).

The straightforward attribution of properties to objects on the orthodox view may be captured by the following two tenets:

(i) Instantiation is absolute.
(ii) Properties are absolute.

A graded metaphysics of properties permits the degreed attribution of properties and, as such, denies that an object having a property is an all-or-nothing matter. There are at least two ways in which the nature of graded qualities may be incorporated into the metaphysical picture: either by denying (i) and integrating degrees into instantiation; or by denying (ii) and providing properties with an internal degreed structure. These possibilities are the *degreed instantiation* approach and the *degreed property* approach, respectively.^3^ I consider each in turn.

### Graded properties as degreed instantiation

As the name suggests, the degreed instantiation approach permits instantiation to come in degrees, hence rejecting tenet (i) of the orthodox view. Furthermore, the account holds on to tenet (ii): properties are absolute. In addition to the ontology of objects and properties on the orthodox view, the instantiation approach further postulates the existence of an instantiation relation that acts as a mediator between the two. Consequently, states of affairs involve triadic relations in which a system *s* and a property *p* stand in an *instantiation relation* to some degree $d_i$. The instantiation relation is graded in the sense that it can have different “strengths" defined in terms of degrees that may take value in the range $0<i\le 1$. The position can be specified as follows:

degreed instantiation: a system *s* has a property *P* to degree $d_i$ if, and only if, *s* instantiates *P* to degree $d_i$, where $0<i\le 1$.

Instantiation to degree $d_i=d_1$ can be interpreted as an object *fully instantiating* the given property, while any $d_i<d_1$ are cases of genuine intermediate instantiation. Notably, *full instantiation* must be distinguished from absolute instantiation of a property as it appears in the orthodox view. Full instantiation is the normalisation of the instantiation relation, by which I mean that instantiation to $d_i=d_1$ is simply the maximum possible instantiation of a given property by an object.^4^ It is reasonable to suppose that the degreed instantiation relation is normalised for each *family* of properties (i.e. properties of the same *kind*), such that a physical system may fully instantiate properties belonging to different families. This ensures that a system may fully instantiate, e.g., the property *scarlet* and the property charge $+e$. Normalisation further ensures that a system may not fully instantiate charge $+e$ and charge $-e$ since these belong to the same property family, namely being *charged*. Nevertheless, although full instantiation is limited in this way, intermediate graded instantiation is possible for several properties belonging to the same family, as long as the total degree of instantiation does not exceed $d_1$.^5^

**Fig. 2:**
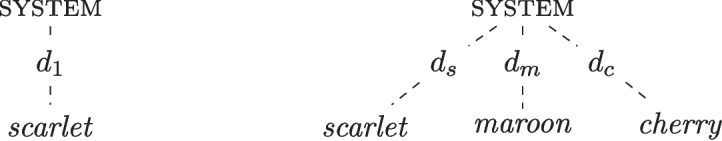
The dashed lines represent degreed instantiation (denoted by $d_1$, $d_{s}$, $d_{m}$, and $d_c$). The system on the left *fully* instantiates the *scarlet*. The system on the right instantiates to some intermediate degree *scarlet*, *maroon*, and *cherry*

Let us consider an example of degreed instantiation. Continuing with the colour example of Fig. 1, an object may instantiate all three properties *scarlet*, *maroon*, and *cherry* (belonging to the *colour* family) to an intermediate degree, as seen in Fig. 2.^6^ The degrees of instantiation must respect normalisation, i.e. $d_s+d_m+d_c \le d_1$. Figure 2 also illustrates the system fully instantiating the colour *scarlet* to degree $d_1$. The system and properties behave as in the orthodox view (i.e. they are absolute), while the instantiation relation provides a graded structure, allowing for more complex property instantiations.

### Graded properties as degreed properties

The degreed property approach is committed to an ontology of objects and properties, and may also admit instantiation relations.^7^ On this account, the gradedness is incorporated directly in the property itself rather than mediated by the instantiation relation. If the account includes an instantiation relation, this relation will, therefore, be absolute, meaning that objects absolutely instantiate degreed properties on this view. This picture provides a dyadic relation wherein a system *s* instantiates a degreed property $P_{d_i}$. In this way, the degreed account adheres to orthodox intuitions that objects either do or do not instantiate (and hence have or do not have) a given property. Instead, the properties diverge from the orthodox view by having an internal graded structure, rejecting tenet (ii). One way to define such a graded property is to think in terms of *collections* of degreed properties: for every $i\in (0,1] \subset \mathbb {R}$ and for every base property *P*, there is a degree $d_i$ such that there exists a degreed property $P_{d_i}$. The set $S_P \ni P_{d_i}$ contains all possible degreed properties defining the degreed property in question.

degreed property: a system *s* has the property *P* to degree $d_i$ if, and only if, *s* instantiates the property $P_{d_i}$, where $P_{d_i}\in S_P$.

Similarly to degreed instantiation, a system *s* has a *full property* *P* if, and only if, the system instantiates the property $P_{d_1}$. Meanwhile, a system can be attributed a given property *P* to an intermediate degree if, and only if, *s* instantiates $P_{d_i}$, where $d_i<d_1$. Once again, it is relevant to distinguish *full properties* from the type of *absolute properties* appearing in the orthodox view; a full property $P_{d_1}$ merely represents the maximum (normalised to degree 1) “strength" with which a system may have the property *P*.

**Fig. 3:**
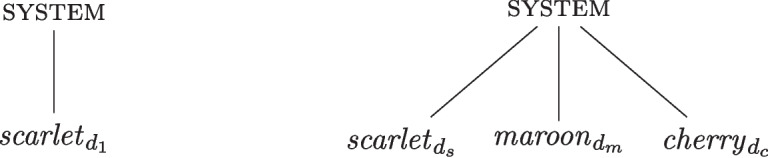
The system on the left absolutely instantiates the *full* (degree 1) property *scarlet*$_{d_1} \in S_{scarlet}$. The system on the right absolutely instantiates the degreed properties *scarlet*$_{d_s}$, *maroon*$_{d_m}$, and *cherry*$_{d_c}$

For illustration, consider once again the example of a system instantiating the colour *scarlet*. In this case, *scarlet* is not an absolute property but is instead represented by a set, $S_{scarlet}$, such that for every $i\in (0,1] \subset \mathbb {R}$ there is a property *scarlet*$_{d_i}\in S_{scarlet}$. Assuming that a system instantiates the property *full scarlet*, the state of affairs is illustrated in Fig. 3. This figure also illustrates the case in which the system can be attributed both *scarlet*, *maroon*, and *cherry* to some intermediate degree. The following three degreed properties are here absolutely instantiated: *scarlet*$_{d_s}$, *maroon*$_{d_m}$, and *cherry*$_{d_c}$, such that the system has the property *scarlet* to degree $d_s$, the property *maroon* to degree $d_m$, and the property *cherry* to degree $d_c$.

As in the case of degreed instantiation, I assume that the normalisation of graded properties is restricted to a single family of properties (for instance, being *coloured* or being *charged*), resulting in the following requirement: $d_s+d_m+d_c \le d_1$. That is, within the property family *coloured* (and in this instance, more specifically, the family *red*), a system cannot have all three properties *scarlet*, *maroon*, and *cherry* to degree $d_1$; absolute instantiation of more than one property within the same family must be the instantiation of degreed properties with an intermediate degree. On the other hand, it should be possible for a system to absolutely instantiate *full* properties from different property families, such that a system instantiating both *scarlet*$_{d_1}$ and $+e_{d_1}$ and may be said to have both the properties *scarlet* and charge $+e$ to degree 1.

The two approaches described in this section succeed in accounting for the graded attribution of properties by supplementing either the instantiation relation or properties with significantly more internal structure than the orthodox view. These views thus reject either tenet (i) or (ii). While the graded property approaches have greater metaphysical commitments than the orthodox view, they also provide greater explanatory power – and result in vastly different metaphysical pictures. This will become clear in the following sections, where I apply these graded views to account for quantum properties.

## Graded locative properties in quantum mechanics

The nature of quantum phenomena has been one of the main motivations for taking a graded metaphysics of properties seriously. In particular, superposition states in quantum mechanics suggest a worldly interpretation in which states of affairs are not an *all-or-nothing* matter as they would be in the orthodox view. Quantum mechanics reveals a microscopic realm with an inherent fuzziness that sharp classical notions cannot easily capture, pointing towards the need for a graded metaphysics (Myrvold, 2017; Ney, 2020).

In this section, I account for this graded nature of location in the quantum domain by applying both the degreed instantiation and degreed approaches to instances of quantum position indefiniteness. More specifically, I explore and develop in detail these graded accounts of location via a quantum mechanical case study, which is presently introduced.

The arguments of this section only presuppose the minimal structure of unitary quantum mechanics.^8^ Discussions of quantum indeterminacy are often carried out in the context of so-called orthodox quantum mechanics, which is unitary quantum mechanics with the addition of a collapse postulate and the eigenstate-eigenvalue link. Section 2.1 uses orthodox quantum mechanics and, in particular, the eigenstate-eigenvalue link to explain why some have taken indeterminacy to arise in quantum mechanics. As mentioned above, however, orthodox quantum mechanics is not required for the arguments of the paper to go through.

### Case study: the electron in a box

In quantum mechanics, observables, represented by self-adjoint operators, denote properties while their corresponding eigenstates represent the possible values of this property. This is often expressed by the eigenstate-eigenvalue link:

eigenstate-eigenvalue link (EEL): A quantum system has a value *v* of an observable property *O*, represented by a self-adjoint operator $\hat{O}$, if and only if its state vector is an eigenstate of that operator with eigenvalue *v*.

In the case of location, the position operator $\hat{X}$ represents the relevant observable in question. However, since the position eigenstates $|{x_i}\rangle$ do not represent possible states, the position operator is instead rewritten in terms of projection operators, $\hat{P}_{r_i}$, associated with spatial regions $r_i$. Here, $\hat{P}_{r_i}|{\psi }\rangle =|{\psi }\rangle$ signifies that the system in state $|{\psi }\rangle$ is in an eigenstate state of $\hat{P}_{r_i}$, meaning that the system can be attributed a definite position at the region $r_i$. In this specific case, the orthodox view can capture the attribution of quantum properties: the system is. However, challenges arise when a system is in a superposition of eigenstates belonging to several projection operators.

**Fig. 4:**
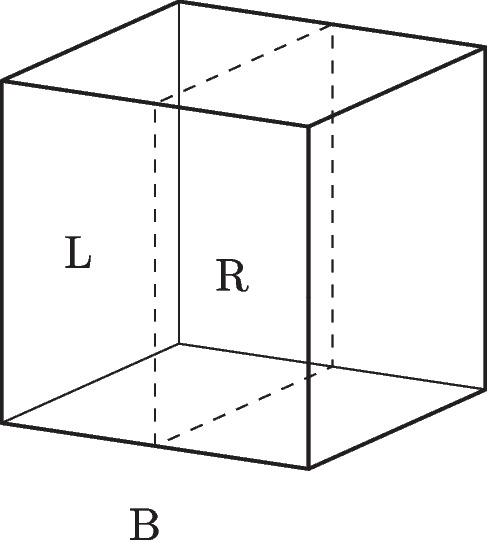
An electron is contained within an (ideally) isolated box *B*. The box is divided into a left and right half denoted *L* and *R*

Let us consider a simple example: an electron in an ideally isolated box. The box *B* can be divided into a left half *L* and a right half *R*, as illustrated in Fig. 4. The following superposition represents the state of the electron:

1 $$\begin{aligned} |{\psi }\rangle = c_L |L\rangle + c_R | R\rangle , \end{aligned}$$

where $| L\rangle$ is an eigenstate of the projection operator $\hat{P}_L$ and the state $| R\rangle$ is an eigenstate of the projection operator $\hat{P}_R$, representing the electron being located at the left-hand and right-hand side of the box respectively, and the complex numbers $c_L$ and $c_R$ are the weighted coefficients of the superposition. Since the system is not in an eigenstate of either of the projection operators, $\hat{P}_L$ and $\hat{P}_R$, it cannot be attributed a definite location at either side of the box according to the eigenstate-eigenvalue link.

In the orthodox view of properties, location is an absolute matter. As such, the orthodox view is unable to attribute any location to the electron in superposition state Eq. 1. Some accounts of location in the quantum domain, such as Pashby (2016) and Glick (2021), attempt to retain the orthodox view of properties and argue that, at most, the electron can be attributed the property of being located at the region corresponding to *B*, leaving it unsettled whether the electron is in the left-hand or right-hand side of the box. However, as argued by (Nørgaard, 2025), these accounts either (i) fail to account for the locative properties of most quantum systems or (ii) conflate the locative properties of systems with different quantum states. This approach omits significant facts about how systems may occupy the same region in potentially vastly dissimilar ways; for instance, it assigns an electron in superposition $|{\psi '}\rangle = c'_L|{L}\rangle +c'_R|{R}\rangle$ the same location as the electron in superposition Eq. 1, namely *B*, even when $c_L\ne c'_L$ and $c_R\ne c'_R$. Hence, the orthodox approach to quantum properties fails to provide a nuanced account of location in the quantum domain.

In the remainder of this section, I investigate two graded accounts of quantum location, which attempt to overcome the shortcomings of the orthodox view by going beyond it. First, I consider how a graded account holding onto the traditional notion of *exact location* may describe graded location; and second, I consider how *quantum location* (Nørgaard, 2025) approaches the issue of graded location in quantum mechanics.

### Graded *exact location*

In the metaphysics of location, *exact location* plays a crucial role in accounting for both the spatial and temporal properties of classical objects (Gilmore, 2008; Gibson & Pooley, 2006; Parson, 2007; Sattig, 2021). *Exact location* is often taken to be the primitive notion from which all other locative notions are defined. Informally, the notion is frequently characterised either in terms of the boundaries of an object (Casati & Varzi, 1999) or with reference to the geometric properties of an object and the region it occupies (Gilmore, 2006):

exact location: system *s* is *exactly located* at a region *r*, *s*@*r*, if, and only if, *s* and *r* have the same shape and size and stand in all the same spatiotemporal relations to other regions and objects.

A feature often attributed to *exact location* is the principle of functionality, which prohibits the possibility of exact multilocation by maintaining that a system has at most one *exact location* at a given time. Following Pashby (2016); Calosi (2021), I adopt this principle throughout the paper and assume that quantum systems cannot be exactly multilocated at one instant of time. In the traditional metaphysics of location, *exact location* is compatible with the orthodox view of properties; if the *exact location* of an object selects a unique region, then for any given region, that object either can or cannot be attributed the property of being *exactly located* there. In the case of quantum mechanics, however, it is not clear that systems such as the electron in state Eq. 1 can be attributed a single region of *exact location* without omitting consequential locative information, as mentioned above. Where the orthodox view must remain silent concerning whether or not the electron is in the left-hand or right-hand side of the box, a graded account of *exact location* may circumvent this difficulty by assigning it an *exact location* at both *L* and *R* to some degree.

As explored in Section 1, a graded account of properties may either incorporate degrees into the instantiation relation or into the properties themselves. A degreed view of quantum properties has been developed in a series of papers by Wilson (2013); Calosi and Wilson (2019, 2021),^9^ focusing on the graded instantiation approach to graded location. In this account, the electron in the box instantiates both possible location properties simultaneously: it instantiates both being *located* at the region *L* and being *located* at the region *R* to a certain degree. For the purposes of this section, I consider the specific case of *exact location*.^10^ How is the degreed instantiation of *exact location* to be specified? The degreed instantiation of a quantum property for a given system is determined by the degree theoretic version of the eigenstate-eigenvalue link (Calosi and Wilson, 2021, p.12):

degree eigenstate-eigenvalue link: a quantum system *s* has a value *v* for the property *O*, represented by a self-adjoint operator $\hat{O}$, to a degree $d_i$, where $i =\vert c \vert ^2$, if, and only if, *c* is the coefficient of the *v*’s eigenvector in the quantum state.

**Fig. 5:**
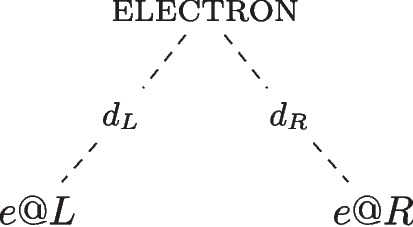
The electron instantiates two properties: being *exactly located* at *L* and being *exactly located* at *R* to the degrees $d_L=d_{\vert c_L \vert ^2}$ and $d_R=d_{\vert c_R \vert ^2}$, respectively

In the case of state Eq. 1, the system instantiates two distinct properties, represented by the superposed states $| L \rangle$ and $| R \rangle$, to degrees specified by the complex coefficients $c_L$ and $c_R$. This means that the electron instantiates to degree $d_L=d_{\vert c_L\vert ^2}$ being *exactly located* at *L* and instantiates to degree $d_R=d_{\vert c_R \vert ^2}$ being *exactly located* at *R*. This locative structure is illustrated in Fig. 5.

The degreed instantiation approach to *exact location* preserves absolute properties as the orthodox view describes them. Since *exact location* is well understood, degreed instantiation provides an ontological picture that is relatively simple: consider the two distinct *absolute* states of affairs “the system is *exactly located* at the region corresponding to the left-hand side of the box" and “the system is *exactly located* at the region corresponding to the right-hand side of the box" (each on their own compatible with the orthodox view). Within each of these individual state of affairs, the property of being *exactly located* is absolute and respects functionality.

However, within the overall state of affairs describing the electron in the superposition state Eq. 1, both of these *exact locations* are instantiated *not absolutely* but to an *intermediate* degree. Hence, the overall state of affairs is incompatible with the absolute nature of the orthodox view of properties. In this way, the graded approach goes beyond the orthodox view and provides a more nuanced description of the location of the electron.

An alternative approach applies degreed properties to provide the backbone of a graded *exact location* account. In this context, the absolute nature of *exact location* is abandoned and replaced instead with a set $S_@$ of *degreed exact locations* $@_{d_i}$, such that for every $i\in (0,1]\subset \mathbb {R}$ there is a *graded exact location* property $@_{d_i} \in S_@$. A concern about this approach is the parting from the absolute nature of *exact location* as usually understood. On this view, the electron in the box has two *degreed exact locations* (belonging to the property set $S_@$) within the same absolute state of affairs, which could potentially create tension with functionality. This is the case if functionalty is taken to deny the absolute instantiation of more than one *degreed exact location*. This reading is justified if being *exactly located* is nothing over and above being *degreed exactly located*. However, on an alternative reading, only full ($d_1$) *degreed exact location* is identified with *exact location*, and here, functionality denies the possibility of the absolute instantiation of more than one full *degreed exact location*. There is no tension with functionality here since the normalisation of the degrees, $\Sigma _i d_i = d_1$, prohibits the possibility of degreed exact multilocation. However, if only full *degreed exact location* is identified with *exact location*, it is unclear to what extent intermediate *degreed exact locations* remain a genuine type of *exact location*, and further metaphysical justification is needed to account for these.^11^

Ultimately, while the degreed property approach may be an option, the degreed instantiation approach better preserves the usual character of *exact location* as an absolute property, incorporating degrees into the instantiation relation, not in the property of *exact location* itself. This preservation makes the degreed instantiation a more natural choice for exploring graded *exact location*.

### Graded *quantum location*

*Quantum location* is a locative notion developed in (Nørgaard, 2025), which provides a graded location account of quantum systems.^12^ While this account utilises degrees, in contrast to graded *exact location*, it is, from the onset, an account of a degreed property.^13^ In other words, the *quantum location* account incorporates the degrees directly *into* the property of being *located* at a region.

The *quantum location account* utilises a localisation scheme associating projection operators $P_{r_i}$ to achronal regions $r_i$ and determining the degree of location at these regions using the Born probabilities. For this discussion, I restrict attention to the location of systems in pure states.

quantum location: a quantum system *s* in state $|{\psi }\rangle$ is *quantum located* at the region *r* to degree $d_i$, $s @_{d_i}^{q} r$, if and only if (i) $p(r) = \langle {\psi }| \hat{P_{r}} |{\psi }\rangle = i$, and (ii) there is no subregion $r' \le r$ such that $p(r') = \langle {\psi }| \hat{P_{r'}} |{\psi }\rangle = i$.

Here, the Born rule provides the probability *p*(*r*) , and the degree $d_i$ respects $0 < i \le 1$. In this way, *quantum location* is a graded property that specifies the degree to which a quantum system is located at a given region. If this metaphysical picture includes an instantiation relation, this must be absolute: a quantum object either does or does not absolutely instantiate the property of being *quantum located* to a degree at a given region. Using the degreed formalism, *quantum location* is represented by a family of degreed properties such that for every $i\in (0,1] \subset \mathbb {R}$ and the base property *quantum location* ($@^q$), there is a degree $d_i$ such that there exists the degreed property $@^q_{d_i} \in S_{@^q}$. Note, however, that the “base property" $@^q$ is not meaningful in and of itself since *quantum location* at a region is always defined in terms of a degree and a system can never be *quantum located* at a region simpliciter. *Quantum location* is therefore straightforwardly defined by the set $@^q_{d_i} \in S_{@^q}$ of continuous elements. This feature of *quantum location* sets it apart from *exact location* and makes the degreed approach ideal for the former and ill-suited for the latter.

**Fig. 6:**
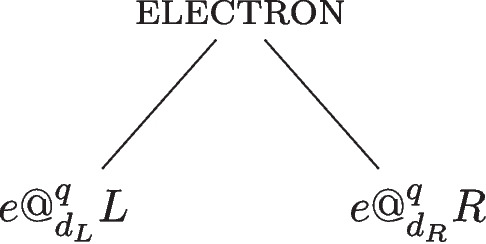
The electron *absolutely* instantiates the properties: being *quantum located* to degree $d_L$ at *L* and being *quantum located* to degree $d_R$ at *R*, where $d_L={\vert c_L \vert ^2}$ and $d_R={\vert c_R \vert ^2}$, respectively

Returning to the electron in a box example, how does *quantum location* account for the system in the superposed state Eq. 1? The Born rule determines the degree of *quantum location* of the system in the left and right half of the box, and the *quantum location* approach picks out the following degree of location: $d_L = d_{\vert c_L \vert ^2}$ and $d_R = d_{\vert c_R \vert ^2}$. Hence, the electron can be assigned the following locative properties: the electron is *quantum located* to degree $d_L$ at the region *L*, and it is *quantum located* to degree $d_{R}$ at the region *R*. The *quantum location* properties of the system are illustrated in Fig. 6.

Furthermore, the *quantum locations* of the system can also be evaluated for different partitions of the box. The electron is *quantum located* to degree $d_1$ at the box *B* itself, $e@^q_{d_1}B$, since the probability of finding the system within this region is $\langle {\psi }| \hat{P}_{B} |{\psi }\rangle =1$, where $\hat{P}_{B}$ represents the projection operator associated with the system being at the region *B*. Since *quantum location* is a degreed property and not an *all-or-nothing* matter, the electron is not simply *located in B*, but *quantum located* to $d_1$ at *B* ($e@_{d_1}B$). Since the degree of *quantum location* at a region is defined by the Born probabilities, it is important to note that, for any given partition, the degree of *quantum location* at all regions must sum to unity. This is a consequence of the normalisation of quantum probabilities, which ensures, for example, that the quantum system with certainty is located in the region, *U*, corresponding to the universe: $1= p(U) \langle { \psi }| \hat{P}_{U} |{\psi }\rangle$. For the electron in the box *B*, this means that $\vert c_L \vert ^2 + \vert c_R \vert ^2 = 1$, since the electron is contained within the boundaries of the perfectly isolated box.

**Fig. 7:**
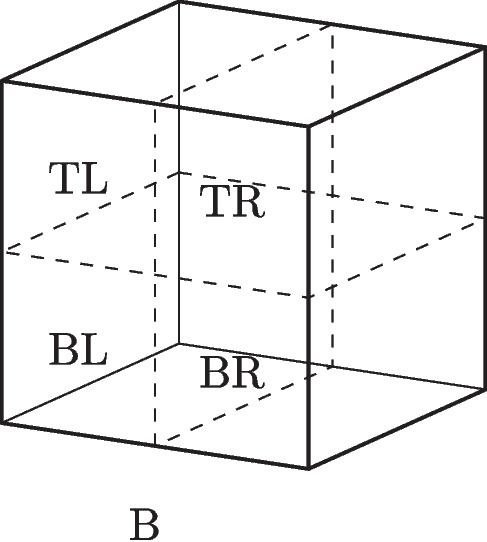
The box *B* can be divided into four quarters, and the quantum state can be rewritten as a superposition of being located at these subregions

This feature generalises: if the box is further divided into quarters, as illustrated in Fig. 7, *quantum location* accounts for the location of the electron at these regions, and the degrees associated with the *quantum location* respect normalisation. For an electron in a superposition state over the top and bottom left half, $|{TL}\rangle$ and $|{BL}\rangle$, as well as the top and bottom right half, $|{TR}\rangle$ and $|{BR}\rangle$:

2 $$\begin{aligned} |{\psi }\rangle = c_{TL} |{TL}\rangle + c_{BL} |{BL}\rangle + c_{TR} |{TR}\rangle + c_{BR} |{BR}\rangle . \end{aligned}$$

The coefficients, and hence degrees of *quantum location* at the relevant subregions of the box, are such that $\vert c_{TL} \vert ^2 +\vert c_{BL} \vert ^2 + \vert c_{TR} \vert ^2 + \vert c_{BR} \vert ^2 =1$. Hence, the electron can be attributed the following locative properties:

$$e@^q_{d_{TL}}TL \wedge e@^q_{d_{BL}}BL \wedge e@^q_{d_{TR}}TR \wedge e@^q_{d_{BR}}BR$$

Here, the explanatory power of the graded nature of *quantum location* becomes apparent: it specifies the locative properties at all subregions of the box, capturing the locative “quantum footprint” of the electron.

Although *quantum location* may, in principle, also be spelt out in terms of the graded instantiation approach, I take it that such an account is overly metaphysically costly since it includes degrees in both the instantiation relation and in *quantum location* itself. Therefore, I do not consider such account in this paper. For now, it is enough to accept that *quantum location* is straightforwardly interpreted as a degreed property.

In this section, two coherent graded accounts of location in the quantum domain have been laid out: the degreed instantiation account of *exact location* and the degreed *quantum location* account. Both approaches reject the absoluteness of the orthodox view by including degrees in the attribution of properties to systems. However, the two accounts presented in this section provide significantly different metaphysical pictures of graded location in quantum mechanics (see Table 1) and hence have different metaphysical implications. In the following section, I investigate how these differences lead to diverging judgements of indeterminacy under two distinct understandings of metaphysical indeterminacy.

**Table 1:** A comparison of the three approaches regarding to their absolute and degreed structure

Account	Locative notion	Instantiation	Properties
Orthodox view	Neutral	Absolute	Absolute
Degreed instantiation of *exact location*	@	Degreed	Absolute
Degreed *quantum location*	$@^q_{d_i}$	Absolute	Degreed

## Quantum indeterminacy: a matter of degree?

It has been argued that a graded metaphysics implies a worldly, or metaphysical, indeterminacy (Rosen & Smith, 2004). In other words, a metaphysics of properties that diverges from the *orthodox view* by allowing intermediate degrees describes a vague or incomplete world in which properties or states of affairs are not entirely definite. Equivalently, it is generally considered the case that a system definitely has a property *P* if, and only if, the system absolutely (or *fully*) instantiates this property *P* and *P* itself is maximally specific and absolute (or *full*). However, in the indeterminacy debate, most of the emphasis has been placed on cases of graded properties as *degreed instantiation* of absolute properties rather than absolute instantiation of *degreed properties*, and the latter approach remains underdeveloped.

In this section, I argue that a graded metaphysics of quantum properties does not necessitate a commitment to quantum metaphysical indeterminacy. I accomplish this by showing that under two distinct interpretations of metaphysical indeterminacy, the *truth-value gap* account (Torza, 2020; Fletcher & Taylor, 2021) and the *determinable-based* account Wilson 2013; Calosi and Wilson 2019; Calosi 2021, degreed instantiation of *exact location* gives rise to metaphysical indeterminacy while the degreed property of *quantum location* does not. Consequently, degrees and indeterminacy come apart: quantum indeterminacy becomes a consequence of one’s approach to graded metaphysics.

### Quantum indeterminacy in the truth-value gap view

Several accounts consider a *truth-value gap*, or *truth-valuelessness*, for a proposition *p* to be indicative of metaphysical indeterminacy. They argue that an account of indeterminacy must let go of the principle of bivalence, i.e. there are propositions that are neither *true* nor *false*, and these represent metaphysically indeterminate states of affairs (Torza, 2020; Fletcher & Taylor, 2021). For instance, for an electron in a superposition state with respect to its spin-*z*, $1/{\sqrt{2}} (|{\uparrow }\rangle _z + |{\downarrow }\rangle _z )$, the proposition p = “the electron has value spin-up in the *z*-direction" on this view would count as neither true nor false and therefore as an example of a truth-valueless sentence. According to the truth-value gap account, the electron in a superposition state presents a metaphysically indeterminate state of affairs, since it is indeterminate whether the electron can be attributed the property of spin-up and spin-down in the *z*-direction. In contrast, proposition *p* would have truth-value 1 if the electron was in the state $|{\uparrow }\rangle _z$, and truth-value 0, if the electron was in state $|{\downarrow }\rangle _z$.

**Fig. 8:**
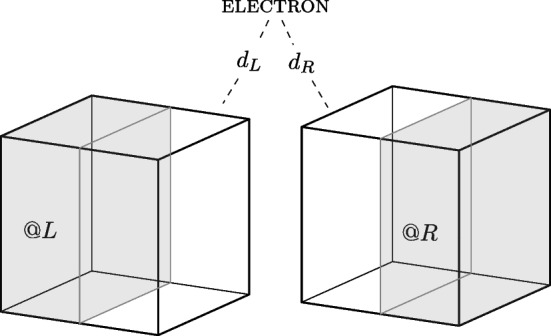
A representation of the electron instantiating the two maximally specific (exhaustive and mutually exclusive) states of affairs: "*e*@*L*" is instantiated to degree $d_L$ and "*e*@*R*" is instantiated to degree $d_R$

#### Degreed instantiation of *exact location*

If the location of the electron is accounted for in terms of the graded instantiation of *exact location*, the electron is indeterminate with respect to its location in the *truth-value gap* interpretation of indeterminacy. On this view, provided the quantum state Eq. 1 and the principle of functionality for absolute *exact location*, there are two exhaustive and mutually exclusive propositions concerning the position of the electron (illustrated in Fig. 8):

$a=$ *e*@*L*, or alternatively: "the electron is *exactly located* at *L*"

$$ b =$$ *e*@*R*, or alternatively: "the electron is *exactly located* at *R*"

Here, the propositions are constructed such that the electron has a unique *exact location* in each. These are exhaustive since both, considered in isolation, describe maximally specific states of affairs of the electron and its location. They are furthermore mutually exclusive since $a\wedge b$ entails that the electron is absolutely *exactly located* at both the regions *L* and *R*, in tension with the principle of functionality.

Given that the electron instantiates both *e*@*L* to degree $d_L$ and *e*@*R* to degree $d_R$, neither of the propositions can be evaluated as true since this would require that either *e*@*L* or *e*@*R* is *fully* instantiated to degree 1; ditto for their falsity. Hence, *a* and *b* are *truth valueless*, and the electron in a box is metaphysical indeterminate with respect to its location.

#### Degreed *quantum location*

**Fig. 9:**
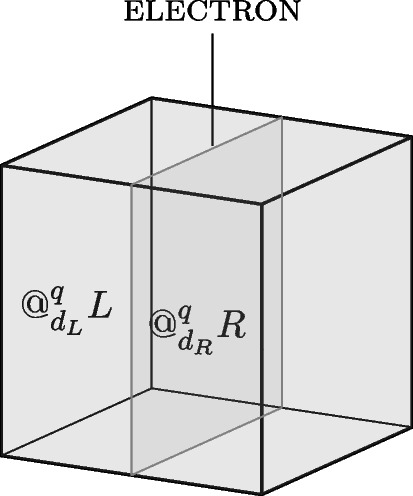
The definite state of affairs of the electron being *degree* $d_L$ *quantum located* at region *L* and *degree* $d_R$ *quantum located* at region *R*

In the case of graded *quantum location*, i.e. the *absolute* instantiation of the degreed property *quantum location*, the electron is evaluated as determinate on the *truth-value gap* interpretation of indeterminacy. This is because the electron absolutely instantiates different degreed *quantum locations* at two distinct regions, such that the following proposition has truth-value 1 (see Fig. 9):

$c= e@^q_{d_L}L \wedge e@^q_{d_R}R$, or alternatively: “the electron is *degree* $d_L$ *quantum located* at the region *L* and *degree* $d_R$ *quantum located* at the region *R*".

Notice that there is no objection to the system being quantum located at two distinct regions since there is no functionality principle for *quantum location*. The only requirement is that the two degrees must respect the normalisation criterion, i.e., the total degree of *quantum location* does not exceed degree 1: $d_L+d_R\le d_1$. Given that the proposition *c* has truth-value 1, there is no indeterminacy on the truth-value gap account.

### Quantum indeterminacy in the determinable-based view

On the other hand, supporters of the determinable-based account of indeterminacy deny that indeterminacy implies a truth-value gap. The determinable-based approach does not involve a truth-value gap because it insists that there are three, rather than two, possible states of affairs involving *p*: the case that *p*, the case that not-*p*, and the case that *p* to some degree, where the latter possibility cannot be reduced to the former two.^14^ According to the determinable-based view, the following proposition should be evaluated when studying the location of the electron:

$d=$“the electron instantiates to degree $d_L$ being *exactly located* at *L* and instantiates to degree $d_R$ being *exactly located* at *R*"

**Fig. 10:**
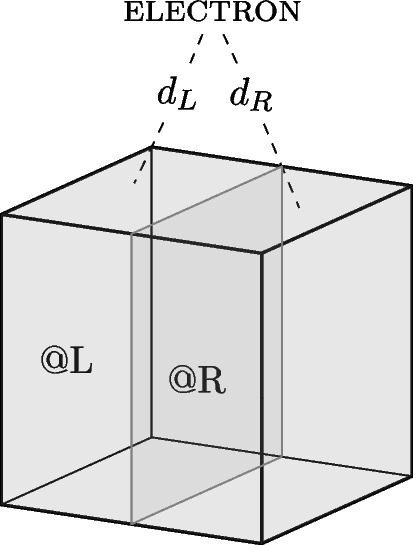
This figure illustrates the maximally specific state of affairs: $d=$ "the electron instantiates being *exactly located* at *L* (@*L*) to degree $d_L$, and the electron instantiates being *exactly located* at *R* (@*R*) to degree $d_R$", which has truth-value 1

The proposition *d* has truth-value 1, and the states of affairs that are degree instantiated are illustrated in Fig. 10.

**Fig. 11:**
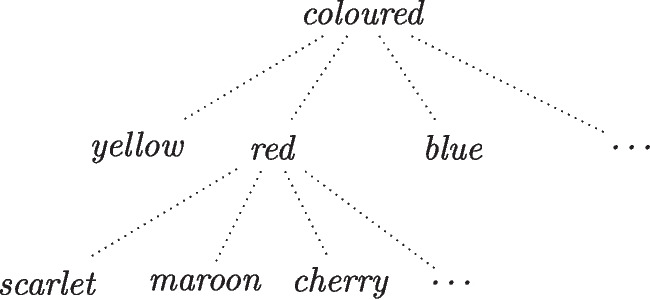
A determinable-determinate structure, which is read top-down. The top levels are lower levels of property specification while the bottom levels hold higher levels of specification determinates

The determinable-based account of indeterminacy works within a determinable-determinate framework, in which properties can be thought of as a hierarchy of determinate specification: any determinable property, like *colour*, can be specified into further determinate properties, like *red*, *blue*, or *yellow*, which themselves are determinables further specifiable in terms of more fine-grained determinates: *red* is then a determinate of *colour* and also a determinable property with determinates including *scarlet*, *cherry*, and *maroon*. In this framework, *red* and *scarlet* are determinates of the same type of determinable property, namely being *coloured*, but are defined at different levels of specification, such that being *scarlet* is a more specific way a system can be *coloured* than being *red* is (see Fig. 11). The lower levels of the hierarchy contain determinates of a higher level of specification, which imply all higher levels of the hierarchy: being *scarlet* implies being *red* and *coloured* as well, a principle known as *determinable inheritance* (Wilson, 2023). Furthermore, each level of specification contains several complete and incompatible determinates, each of which can be instantiated on its own and only one of which can be absolutely attributed to a system at any given time on the orthodox view; for instance, a system cannot absolutely instantiate both absolute properties *scarlet* and *cherry* simultaneously.

The determinable-based account attributes the indeterminacy of a system, with respect to a given property, to the failure of the unique attribution of a determinate for the determinable property in question. Wilson (2013, p.366) defines metaphysical indeterminacy as follows:

What it is for a [state of affairs] to be [metaphysically indeterminate] in a given respect R at a time *t* is for the [state of affairs] to constitutively involve an object (more generally, entity) *O* such that (i) *O* has a determinable property *P* at *t*, and (ii) for some level *L* of determination of *P*, *O* does not have a unique level-*L* determinate of *P* at *t*.

An entity *O* can fail to have a unique level-*L* determinate of a property *P* by either being *gappy* (having no determinate at some level-*L* of specification) or *glutty* (having more than one level-*L* determinate). Here, *glutty* indeterminacy is most often expressed in terms of degrees, such that if the entity in question has *more than one* level-*L* determinate of *P*, to some intermediate degree $d_i<d_1$, then the entity is indeterminate with respect to the property *P*.^15^

To evaluate whether or not the graded quantum property accounts (from Section 2) are indeterminate, the following question must be answered: what are the determinates of the degree instantiated *exact location* and the degreed *quantum location* approach? Before turning to this question, it is worth reviewing *non-graded* determinables, determinates, and specification levels in quantum mechanics.

#### Quantum determinates and specification levels

Quantum observables can be identified with the maximally unspecific determinable properties of a system; as already established, for quantum systems, the position operator $\hat{X}$ represents the maximally unspecific determinable property of location. Determinables of a higher level of specification are constructed by more fine-grained projective decompositions. In the case of location, the position operator is fine-grained by decomposing the operator into the following projection operators:

3 $$\begin{aligned} \hat{X}=\sum _j x_i \hat{P}_{r_j}, \end{aligned}$$

where $r_j$ are disjoint regions whose mereological union composes all of space, and $x_i$ are the eigenvalues. Here, the projection operators represent determinate properties of the maximally unspecific determinable represented by the position operator. If a system is in an eigenstate $|{r}\rangle$, corresponding to the eigenstate of projection operator $\hat{P}_{r}$, it can be attributed a determinate location at the region *r*. In this way, the specification levels of location are represented by fine-grainings of the position operator. The smaller the regions of fine-graining, the higher the specification of the location: for instance, being *located at the region B* is a determinate property with a higher level of specification than the determinable property of being *located in space*.

The location of a quantum system represented by state $| \psi \rangle$, at a given level of specification, is evaluated by writing the state as a sum of the eigenstates associated with the spectral decomposition of the position operator at this level:

4 $$\begin{aligned} | \psi \rangle = \sum _{i} c_i |{r_i}\rangle , \end{aligned}$$

where $|{r_i}\rangle$ is an eigenstate of the projection operator $P_{r_i}$ associated with the region $r_i$; note that Eq. 4 is the application of the projective decomposition Eq. 3 to the quantum state $|{\psi }\rangle$. The superposition state Eq. 1 is one such decomposition, where the kets $| L \rangle$ and $| R \rangle$ represent the eigenstates of projection operators associated with the regions *L* and *R*. This represents a specification level, call it level-(1), with locations at *L* and *R* representing the two determinates for this level of specification. Meanwhile, the superposition state Eq. 2 is another such decomposition, providing a more fine-grained representation of the quantum state than the superposition Eq. 1. Similarly, level-(*B*) is represented by the decomposed state:

5 $$\begin{aligned} |{\psi }\rangle = c_B|{B}\rangle , \end{aligned}$$

where $|{B}\rangle$ corresponds to the entire region of the box and $c_B=1$. Level-(*B*) is a lower level of specification than both level-(1) and level-(2), i.e. level-(1) and level-(2) are *more fine-grained* than level-(*B*). In this way, the choice of decomposition basis determines the level of specification, such that a basis of eigenstates belonging to projection operators for smaller regions are determinates of a higher level of specification than the determinates from projection operators associated with larger regions.^16^

Following Calosi (2021), I will use $\text{d}^{(j)}$ to represent a determinate property at the *j*th specification level and stipulate that $\text{d}^{(1)}$ is specified by the basis states in Eq. 1, and $\text{d}^{(B)}$ is a determinate at level-(*B*) specification represented by Eq. 5. Here, $\text{d}^{(B)}$ are determinates at a lower level of specification than determinates $\text{d}^{(1)}$, since the decomposed state Eq. 1 maps onto smaller regions within the box *B* than the state Eq. 5.

#### Degreed instantiation of *exact location*

Let us now go back to the issue of quantum indeterminacy and graded *exact location*: what are the *exact location* determinates of the electron in the box? Consider once again the superposition state Eq. 1, denoting level-(1) specification. Since *exact location* is an absolute property on this account, and it is the instantiation relation that admits degrees, it is natural to think that in Eq. 1, the eigenstates represent the determinates, while (the square modulus of) the complex coefficients represent their degree of instantiation:

$$\begin{aligned} | \psi \rangle = \underbrace{c_L}_{d_L}\underbrace{| L \rangle }_{\text {d}^{(1)}_L} + \underbrace{c_R}_{d_R}\underbrace{| R \rangle }_{\text {d}^{(1)}_R} \end{aligned}$$

In this case, $|L\rangle$ and $|R \rangle$ represent the determinates $\text{d}^{(1)}_L$ and $\text{d}^{(1)}_R$, which are instantiated to degrees $d_L$ and $d_R$ respectively. Hence, being *exactly located* at the region *L* and being *exactly located* at the region *R* are the relevant determinates of a quantum system. The determinates are at the same level of specification since they appear in the decomposition basis of Eq. 1. Given that the electron instantiates the two same-level determinates @*L* and @*R* to an intermediate degree, this is a case of *glutty* instantiation; the system has no unique level-(1) determinate. Hence, the electron is indeterminate with respect to its *exact location* on the *determinable-based* interpretation of indeterminacy.

It is important to note that this conclusion depends on a certain choice of maximally specific determinate level. In the non-graded classical case, *exact location* can be considered as a maximally specific determinate of determinable *entire* or *weak location*. If the same approach is applied in the quantum case, then the determinates of the maximally specific level will be *exact locations* of the system, while all lower specification levels are *entire locations*. For classical objects, maximally specific determinates correspond to regions with the same shape and size as the object; however, for the electron in the box, it is not clear which regions of location represent maximally specific determinates. If decompositions of the quantum state represent possible specification levels, there is no physically preferred specification level: the arbitrariness of basis choice entails that the same quantum state can be decomposed in infinitely many ways. However, there may be two metaphysically natural candidates for the maximally specific level of the electron in the box: (i) the specification level that ensures instantiation of a unique determinate, namely level-(*B*) (Glick, 2017), or (ii) the level with the smallest possible regions of entire location (Calosi, 2019).^17^ On option (i), the electron instantiates a unique level-(*B*) determinate: since $\vert c_B\vert ^2=1$ the electron instantiates to degree $d_1$ being *exactly located* at the region corresponding to the box (*e*@*B*), and indeterminacy does not arise. On option (ii), the maximally specific determination level is a generalisation of level-(1), and here the electron will instantiate to some degree several determinates, resulting in glutty indeterminacy. Here, graded instantiation results in metaphysical indeterminacy.

Arguably, degreed instantiation is superfluous on option (i) since the electron location may as well be stated in orthodox terms: the electron absolutely instantiates being *exactly located* at *B*. Since the connection between graded instantiation and indeterminacy is of interest here, trivial instances of degreed instantiation, like option (i), are not relevant, and only non-trivial degreed instantiations, like option (ii), should be considered. Indeed, if a determinable-determinate based account of *exact location* in quantum mechanics permits intermediate degrees of instantiation, then the systems will have no unique *exact location* at the maximally specific level of specification, resulting in glutty indeterminacy with respect to the *exact location* of the system in question.

#### Degreed *quantum location*

How does the *quantum location* account fare on the *determinable-based* interpretation of indeterminacy? To answer this question, it is once again necessary to identify the determinates of *quantum location* for an electron in the state Eq. 1. In contrast to the degreed instantiation of *exact location*, *quantum location* is a degreed property, the determinates of which cannot be represented by eigenstates (of projection operators for the position operator) with the degree appended; the determinates of *quantum location* must contain an internal degree. If the eigenstates are taken to represent the “base property" and the associated complex coefficient represents its degree, two candidates for determinates naturally arise for *quantum location*. For the electron in the state Eq. 1, these candidates are the following:

- the complex coefficient-eigenstate combination, i.e. the eigenstate (of a projection operator) combined with its complex coefficient:
  $$\begin{aligned} |{\psi }\rangle = \underbrace{c_L|{L}\rangle }_{\text {d}^\text {(1)}_{d_L}} + \underbrace{c_R |{R}\rangle }_{\text {d}^{(1)}_{d_R}} \end{aligned}$$
- the linear combination of the coefficient-eigenstate pairs, i.e. the decomposed state in its entirety:
  $$\begin{aligned} |{\psi }\rangle = \underbrace{c_L| L\rangle + c_R | R\rangle }_{\text {d}^{(1)}_{(d_L,d_R)}} \end{aligned}$$

##### Candidate (a)

Candidate (a) takes the combined elements $c_L| L \rangle$ and $c_R| R\rangle$ to represent two determinates of *quantum location*: $\text{d}^{(1)}_{d_L} \equiv @^q_{d_L}L$ and $\text{d}^{(1)}_{d_R} \equiv @^q_{d_R}R$ on specification level-(1). Table 2 shows an example of such determinates. Although natural candidates at first glance, (a) candidates are incomplete and therefore not possible determinates of *quantum location*. This incompleteness is evident in their failure to satisfy *determinable inheritance*, which requires that the attribution of a candidate (a) determinate ensures the attribution of all corresponding lower-level determinable quantum locations. For the electron in the box, this means that the *quantum location* determinates at the level-(1) must ensure the attribution of all higher-level determinables of *quantum locations*, including level-(*B*) properties; in contrast to the *exact location* case, the determinates will be *quantum locations* at all levels of specification, not just at the maximally specific one. However, this determinable inheritance is not satisfied. On its own, the candidate (a) determinate $d^{(1)}_{d_L}$ ($@^q_{d_L}L$) does not ensure the determinable $d^{(B)}_{d_1}$ ($@^q_{d_1}B$) – only the instantiation of both determinates $d^{(1)}_{d_L}$ and $d^{(1)}_{d_R}$ ensures that the system is *quantum located* to degree $d_1$ on the level-(*B*) specification. This means candidate (a) determinates are incomplete and therefore do not represent viable determinates of quantum location.

**Table 2:** Table showing two determinates at the level-(1) specification, with their corresponding label and determinate degreed *quantum location* property

State	$\text{d}^{(1)}_{d_i}$	Determinate
$\frac{1}{\sqrt{2}}\left( |{L}\rangle + |{R}\rangle \right)$	$\text{d}^{(1)}_{d_L=d_{\frac{1}{2}}}$	$@^q_{d_{\frac{1}{2}}}L$
	$\text{d}^{(1)}_{d_R=d_{\frac{1}{2}}}$	$@^q_{d_{\frac{1}{2}}}R$
$\frac{1}{2}|{L}\rangle + \frac{\sqrt{3}}{2}|{R}\rangle$	$\text{d}^{(1)}_{d_L=d_{\frac{1}{4}}}$	$@^q_{d_{\frac{1}{4}}}L$
	$\text{d}^{(1)}_{d_R=d_{\frac{3}{4}}}$	$@^q_{d_{\frac{3}{4}}}R$

**Table 3:** Three examples of quantum states in superposition Eq. 1 (and correspondingly at a level-(1) specification) with their corresponding label and determinate degreed *quantum location* property

State	$\text{d}^{(1)}_{(d_L,d_R)}$	Determinate
$\frac{1}{\sqrt{2}}\left( |{L}\rangle + |{R}\rangle \right)$	$\text{d}^{(1)}_{(d_{\frac{1}{2}},d_{\frac{1}{2}})}$	$@^q_{d_{\frac{1}{2}}}L \wedge @^q_{d_{\frac{1}{2}}}R$
$\frac{1}{2}|{L}\rangle +\frac{\sqrt{3}}{2}|{R}\rangle$	$\text{d}^{(1)}_{(d_{\frac{1}{4}},d_{\frac{3}{4})}}$	$@^q_{d_{\frac{1}{4}}}L \wedge @^q_{d_{\frac{3}{4}}}R$

##### Candidate (b)

Candidate (b) takes the entire decomposed quantum state to represent the relevant determinate for the level of specification provided by the chosen decomposition basis. For the electron in the box, on specification level-(1), the whole of $c_L | L\rangle + c_R | R\rangle$ represents a *single* determinate that is a conjunction of *quantum locations*, namely: $\text{d}^{(1)}_{(d_L,d_R) }\equiv @^q_{d_L}L\wedge @^q_{d_R}R$. Here, the notation $(d_L,d_R)$ is an ordered pair of degrees determined by the coefficients associated with the basis vectors $| L\rangle$ and $| R \rangle$, respectively. The degrees in the ordered pair $(d_L,d_R)$ are such that $d_L$ and $d_R$ can take on any degree $d_i$, with $i \in (0,1] \subset \mathbb {R}$, as long as $d_L+d_R=d_1$, and each such combination represents a determinate of the level-(1) specification. There are just as many determinates of *quantum location*, at specification level-(1) as there are possible values for the ordered set $(d_L,d_R)$; that is, at the level-(1) specification there are an infinite number of determinates. Table 3 shows some example states and their determinates.

**Fig. 12:**
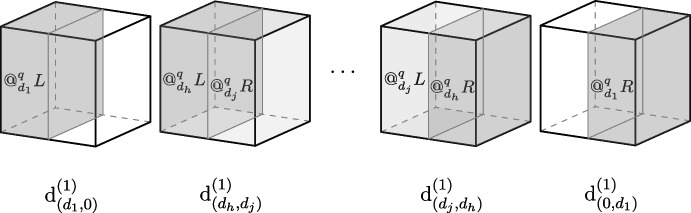
A representation of the possible determinates of *quantum location* for the electron in superposition state Eq. 1 and specification level-(1)

Candidate (b) determinates are in one-to-one correspondence with basis decompositions of quantum states: each possible state specifies a single determinate constructed from a conjunction of *quantum locations*, assigning one specific degreed *quantum location* to each region specified by the decomposition basis. It is important to clarify that the determinates of *quantum location* are *not* identified with quantum states, but rather the conjunction of the *quantum locations* instantiated by a system. Candidate (b) determinates are absolutely attributed complex properties composed of quantum locations at different regions.^18^ These *quantum location* determinates thus represent the absolute instantiation of a collection of graded location properties connecting the quantum system to distinct regions. These determinates represent different incompatible ways in which a system can occupy a region, and for each specification level, there are as many determinates as there are distinguishable states. This fact is illustrated in Fig. 12. There, *h* represents the greatest possible value less than 1, such that $d_h<d_1$ and *j* represents the smallest possible value greater than 0 such that $d_j$ is the smallest possible degree, and $d_h+d_j=d_1$. The resulting picture is an infinite number of determinates for the level-(1) specification of the *quantum location* of the electron in the box.

Since the basis-decomposition of the quantum state of a system *in its entirety* represents the possible determinates of location at a given level of specification, candidate (b) determinates ensure *determinable inheritance*; the decomposition of the quantum state at level-(1) is more fine-grained than level-(*B*), which means that $\text{d}^{(1)_{(d_L,d_R)}}$ implies $\text{d}^{(B)}_{d_1}$. Furthermore, since the quantum state is unique, the system will absolutely instantiate a unique determinate for every level of specification. Consequently, a system may instantiate several graded *quantum locations* without giving rise to indeterminacy. At *every* level of specification on the *determinable-based* account of quantum location, a single determinate is instantiated.

In conclusion, in this section, I have shown that while the degreed instantiation view of *exact location* is evaluated as indeterminate on two influential understandings of metaphysical indeterminacy, the degreed *quantum location* approach gives rise to quantum indeterminacy on neither.

## Concluding remarks

In this paper, I have investigated two approaches to location in quantum mechanics accounting for quantum position indefiniteness in terms of degrees. In Section 1, I presented two distinct approaches to graded properties, the first incorporating degrees in the instantiation relation and preserving orthodox properties, and the second incorporating degrees into the properties themselves abandoning the *all-or-nothing* absolute properties of the orthodox view. In Section 2, I argued that these strategies might be used to account for the graded nature of location in the quantum domain, pairing degreed instantiation with the absolute property *exact location* and arguing that the locative notion *quantum location* is naturally understood as a degreed quantum property. In Section 3, I investigated how these accounts of graded location are evaluated under two influential approaches to quantum metaphysical indeterminacy: the *truth-value* gap and the *determinable-based* approach. While degreed instantiation of *exact location* is indeterminate on both approaches, degreed *quantum location* is indeterminate on neither.

These findings suggest that indeterminacy and degrees are not necessarily linked and that it is possible to provide a graded metaphysics of quantum location that does not entail indeterminacy. *Quantum location* provides a novel account of graded location in quantum mechanics, taking the *inexact* nature of location in the quantum realm seriously. The upshot of this paper is that quantum indeterminacy may emerge as a consequence of holding onto absolute properties familiar from the classical domain, given that indeterminacy arises for graded instantiation of absolute properties but not for the absolute instantiation of graded properties. This motivates a shift in our understanding of quantum properties: embracing a graded view of quantum properties may provide a novel approach to quantum ontology. It remains to be seen whether this strategy may be extended to other quantum properties. Is quantum indeterminacy a consequence of one’s approach to graded metaphysics?

## Data Availability

Not applicable

## Footnotes

1 For a general argument in defence of this connection, see (Rosen & Smith, 2004).

2 I assume a realist reading of properties. In addition, an instantiation relation *may* be posited, acting as a mediator between the two. However, instantiation is often considered a feature of properties rather than a further ontological commitment, and the instantiation relation is not necessary for the orthodox view.

3 The definitions provided in this section agree with those provided by (Calosi & Michels, 2025).

4 Whether or not *fully instantiated* can be identified with *absolute instantiation* is a separate question I will not pursue in this paper.

5 Of course, there is no reason to suppose that an object must necessarily instantiate a specific property or family of properties fully, i.e. there is no reason to suppose that given a particular system, the degrees of instantiation for properties within a specific family must sum to one.

6 I recognise the limitations of the colour example, for while it illustrates degreed instantiation structure, it can be challenging to grasp its intuitive meaning. There are ways in which it should *not* be interpreted. Degreed instantiation should *not* be understood as the system being partly *scarlet*, partly *maroon*, and partly *cherry*. Nor should it be understood in perspective-relative terms as presented by (Wilson, 2013), such that the system is *scarlet* from one perspective, *maroon* from a second, and *cherry* from a third. Instead, the system can be said to *partly* instantiate several absolute properties *scarlet*, *maroon*, and *cherry* simpliciter; while this is unintuitive in a classical setting, it is more natural in a quantum setting with superposition states.

7 While the degreed property approach does not necessitate an ontological commitment to instantiation relations, I include it here for completeness.

8 In particular, following Wallace (2019), I take this to include unitary quantum mechanics plus decoherence plus any further structure needed to account for measurements; which structure is needed for measurement, however, is left unspecified, since none of the arguments of this paper depend on it. I leave the question of its validity in specific interpretations of quantum mechanics to future work.

9 With a focus on location in the double slit experiment in Calosi and Wilson (2021)

10 The determinable-determinate account of degreed location does not specify the locative notion in question. It is natural to assume that *exact location* is implied, but the account may, in principle, utilise any locative notion. In either case, this does not hinder the specific application of the approach to *exact location* in this section.

11 Note that this issue does not arise for the degreed instantiation approach to *exact location*. Here, functionality may be taken to deny more than one full ($d_1$) instantiation of absolute *exact location* (ensured by normalisation). This creates no challenges for intuitions about the nature of *exact location*.

12 I note that the term “quantum location" has been used elsewhere in the literature and that the notion should be kept distinct from uses in (Pashby, 2016) and (Glick, 2021).

13 *Quantum location* is a novel locative notion developed for the quantum realm, entirely distinct from *exact location*. It circumvents the challenges of the *degreed exact location* property since *quantum location* does not rely on *exact location* in any way. I refer to (Nørgaard, 2025) for in-depth arguments in favour of *quantum location* and questions concerning the connection between *quantum location* and the traditional metaphysics of location.

14 Indeed, one may even think that the former two propositions are special instances of the degreed propositions, such that “*it is the case that* *p*" if, and only if, “*it is the case that* a *to degree* $d_1$" and “*it is the case that not-**p*" when *p* fails to be the case to any degree.

15 The *glutty* view of indeterminacy may also be expressed in terms of relative perspectives rather than degrees (Wilson, 2013; Calosi & Wilson, 2019). However, only the degreed view of determinable-based indeterminacy is relevant for discussing graded properties.

16 There is more to be said concerning the determinable-determinate structure of quantum properties in terms of their state decomposition. For instance, some equivalence classes between certain specifications must be implemented to obtain the required semi-lattice structure. For the arguments of this paper, however, it is sufficient to establish that level-(*B*) is a lower level of specification than level-(1).

17 Calosi (2019) works with these maximally specific regions as spatial points; however, the Heisenberg uncertainty principle requires the regions to have some minimal extension.

18 This determinate is complex in two aspects: (i) it has complex conjuncts (constructed from a base property and degrees), and (ii) furthermore constructs a complex determinate property from these. This requires an appropriate metaphysical framework to fill out the formal details, a substantive issue which I leave for future investigation. The informal characterisation provided here should suffice for the scope of this paper.
